# Sensorineural Hearing Loss and Mitochondrial Apoptosis of Cochlear Spiral Ganglion Neurons in Fibroblast Growth Factor 13 Knockout Mice

**DOI:** 10.3389/fncel.2021.658586

**Published:** 2021-06-16

**Authors:** Yulou Yu, Jing Yang, Feng Luan, Guoqiang Gu, Ran Zhao, Qiong Wang, Zishan Dong, Junming Tang, Wei Wang, Jinpeng Sun, Ping Lv, Hailin Zhang, Chuan Wang

**Affiliations:** ^1^The Key Laboratory of Neural and Vascular Biology, Ministry of Education, Hebei Medical University, Shijiazhuang, China; ^2^The Key Laboratory of New Drug Pharmacology and Toxicology, Department of Pharmacology, Hebei Medical University, Shijiazhuang, China; ^3^Department of Physiology, Hebei Medical University, Shijiazhuang, China; ^4^Department of Otolaryngology, The Third Hospital of Hebei Medical University, Shijiazhuang, China; ^5^Department of Cardiology, The Second Hospital of Hebei Medical University, Shijiazhuang, China; ^6^Hubei Key Laboratory of Embryonic Stem Cell Research, Hubei University of Medicine, Shiyan, China; ^7^Key Laboratory Experimental Teratology of the Ministry of Education, Department of Biochemistry and Molecular Biology, School of Basic Medical Sciences, Cheeloo College of Medicine, Shandong University, Jinan, China

**Keywords:** fibroblast growth factor 13, deafness, spiral ganglion neuron, apoptosis, mitochondria, syndrome

## Abstract

Deafness is known to occur in more than 400 syndromes and accounts for almost 30% of hereditary hearing loss. The molecular mechanisms underlying such syndromic deafness remain unclear. Furthermore, deafness has been a common feature in patients with three main syndromes, the BÖrjeson-Forssman-Lehmann syndrome, Wildervanck syndrome, and Congenital Generalized Hirsutism, all of which are characterized by loss-of-function mutations in the *Fgf13* gene. Whether the pathogenesis of deafness in these syndromes is associated with the *Fgf13* mutation is not known. To elucidate its role in auditory function, we generated a mouse line with conditional knockout of the *Fgf13* gene in the inner ear (*Fgf13* cKO). FGF13 is expressed predominantly in the organ of Corti, spiral ganglion neurons (SGNs), stria vascularis, and the supporting cells. Conditional knockout of the gene in the inner ear led to sensorineural deafness with low amplitude and increased latency of wave I in the auditory brainstem response test but had a normal distortion product otoacoustic emission threshold. *Fgf13* deficiency resulted in decreased SGN density from the apical to the basal region without significant morphological changes and those in the number of hair cells. TUNEL and caspase-3 immunocytochemistry assays showed that apoptotic cell death mediated the loss of SGNs. Further detection of apoptotic factors through qRT-PCR suggested the activation of the mitochondrial apoptotic pathway in SGNs. Together, this study reveals a novel role for *Fgf13* in auditory function, and indicates that the gene could be a potential candidate for understanding deafness. These findings may provide new perspectives on the molecular mechanisms and novel therapeutic targets for treatment deafness.

## Introduction

Hearing loss is one of the most common sensory deficits that can occur in newborns and has been linked to many environmental and genetic factors (Morton and Nance, 2006; Ideura et al., 2019). The mammalian cochlea (the peripheral organ for hearing) is a small, yet complicated snail-shaped organ that consists of heterologous cell types arranged precisely to detect and process sounds. Hair cells (HCs) convert the physical vibrations generated by sound stimuli into chemical signals, which are then transmitted by spiral ganglion neurons (SGNs) to the central nervous system via ribbon synapses (Coate and Kelley, 2013; Li et al., 2017). Both conductive and sensorineural impairments have been known to cause hearing loss. Damage to HCs or SGNs often results in irreversible, permanent sensorineural hearing loss due to their limited self-regenerating capacity (Kwon et al., 2014; Wong and Ryan, 2015).

Hearing loss, that is not associated with any other sign and symptom, is designated as non-syndromic hearing loss (NSHL) (Dror and Avraham, 2010), mainly caused due to mutations in the genes that are involved in auditory functions. More than 120 genes have been associated with NSHL^1^. In contrast, hearing loss is known to occur more frequently in syndromes that affect various other systems of the body, referred to as syndromic hearing loss (SHL) (Chen et al., 2016). Currently, there are over 400 syndromes that result in hearing loss, corresponding to 30% of inherited deafness cases (Ideura et al., 2019). Meanwhile, the molecular mechanisms that underlie the pathogenesis of most SHL have not yet been determined, presenting a great challenge to its clinical treatment. Previous studies have shown deafness to be a common feature in patients with the BÖrjeson-Forssman-Lehmann syndrome (BFLS; OMIM #301900), Wildervanck syndrome (WS; OMIM %314600), and Congenital Generalized Hirsutism (CGH; OMIM #307150) (Gecz et al., 1999; DeStefano et al., 2013; Abu-Amero et al., 2014). Interestingly, patients with these syndromes are characterized by a loss-of-function mutation in the *Fgf13* gene. Whether the pathogenesis of deafness in these syndromic patients is associated with the *Fgf13* mutation is unclear.

Fibroblast growth factor (FGF) 13 is a multifunctional non-secretory protein that belongs to the FGF homologous factor (FHF) subfamily, which includes four genes *Fhf1-4* (with the corresponding name *Fgf11*-*Fgf14*) based on distinct alternative sequences (Goldfarb, 2005; Wang et al., 2011, 2017; Yang et al., 2016; Wei et al., 2017). FHFs are involved in modulating voltage-dependent sodium channels (Liu et al., 2003; Lou et al., 2005; Goetz et al., 2009; Wang et al., 2011) and microtubule-stabilizing proteins (Wu et al., 2012). FHFs are widely expressed in the brain and are known to play crucial roles in the development and functioning of the nervous system (Goldfarb, 2005). Disruption of *Fgf13* causes genetic epilepsy and febrile seizures plus (GEFS^+^) syndrome (Puranam et al., 2015), as well as results in impaired learning and memory (Wu et al., 2012). Furthermore, the *Fgf14* mutation has been known to induce inherited ataxia (Dalski et al., 2005), while the role of *Fgf13* in the auditory system has not yet been determined. Increased FGF13 expression has also been linked to cancer progression (Okada et al., 2013; Liu et al., 2018; Song and Li, 2019), and depletion of *Fgf13* induces apoptosis in cancer cells (Bublik et al., 2016). But its role in apoptotic pathways remains unknown.

In this study, we hypothesized *Fgf13* loss of function to be associated with hearing loss. We examined the localization of FGF13 in the murine cochlear tissue. Furthermore, a transgenic mouse line with a conditional knockout of the gene in the inner ear area (*Fgf13* cKO) was generated and characterized using ethological tests. We investigated the morphological structural changes of the cochlea and the potential underlying mechanisms in these mice. We found that *Fgf13* deficiency caused sensorineural deafness with activated mitochondrial apoptotic cell death in SGNs from the apical to the basal region, but there were no significant morphological and number changes in the HCs. Our study revealed a novel role for *Fgf13* in auditory function, where it regulates the survival of SGNs in the inner ear. Our results indicate that the gene could be a potential candidate for deafness, thus providing new insights into understanding its pathogenesis and creating novel therapeutic targets.

## Materials and Methods

### Ethics Statement

All animal experiments were approved by the Laboratory Animal Ethical and Welfare Committee of Hebei Medical University (Shijiazhuang, China, Approval No. IACUC- Hebmu-PD-201720). All procedures were carried out in accordance with the National Institutes of Health Guide for the Care and Use of Laboratory Animals (Tan et al., 2018).

### Animals

All genetically modified mice were maintained on a C57BL/6J genetic background. We generated a mouse line with *Fgf13* conditional knockout in the inner ear via *Cre/loxP*-mediated recombination by mating *Fgf13-loxP* mice (*Fgf13*^fl/fl^ or *Fgf13*^fl/Y^) with Atoh1-cre mice (Tg (Atoh1-cre) 1Bfri, also named Math1-cre, MGI #011104 from the Jackson Laboratory). *Fgf13-loxP* mice were generated in collaboration with Beijing Biocytogen, Co., Ltd. (Beijing, China) by flanking exon 3 of the mouse *Fgf13* gene with two *loxP* sites as described previously (Wang et al., 2017). Genotypes were verified by PCR. Specifically, Genomic DNA was extracted from the mouse tail tip using the following specific primers:

PCR for identification of *Fgf13-loxP* gene fragment: 5′- TAGTTCCATCTAACAGGGCTCATG (forward) and 5′- AGA CTTTGGTGGGAGCATCCTG (reverse). PCR for identification of *Fgf13 Frt* gene fragment: 5′- AGTTCGACAGACAGTGCCA TTG (forward) and 5′- TCTGAACAGATTAGTAATGAACACA GATG (reverse). PCR for identification of *Atoh1-Cre* gene fragment: 5′- CCGGCAGAGTTTACAGAAGC (forward) and 5′- CTAGGCCACAGAATTGAAAGATCT (reverse). PCR for identification of *Atoh1-Cre* control gene fragment: 5′- GTAGGTGGAAATTCTAGCATCATCC (forward) and 5′- ATGTTTAGCTGG CCCAAATG (reverse).

The sizes of amplified PCR products were 241 bp for the *loxP* allele or 183 bp for the wild-type (WT) allele; 324 bp for the *Cre* control allele and 450 bp for the *Cre* allele. Homozygous mice *Fgf13*^fl/Y^; *Atoh1-cre* (Fgf13^–/Y^) and *Fgf13*^–/–^; *Atoh1-cre* (*Fgf13*^–/–^) were denoted knockout (*Fgf13* cKO) mice and *Fgf13*^–/+^; *Atoh1-cre* (*Fgf13*^–/+^) were heterozygous knockout mice. WT and Atoh1-cre mice were used as the control mice. All animals (males and females) used in our experiments were adult C57BL/6J mice aged 8–12 weeks.

### Assessment of Auditory Functions

Auditory brainstem recording (ABR) and distortion product otoacoustic emission (DPOAE) recordings were performed in WT, Atoh1-cre and *Fgf13* cKO mice in a soundproof room. Mice were anesthetized with intraperitoneal injections of 10% chloral hydrate (0.04 mL/10 g body weight, dissolved in saline solution) before recordings. The depth of anesthesia was periodically verified by the lack of foot-pinch response. For ABR recordings (Guarch et al., 2018), three needle electrodes were inserted sub-dermally at the cranial vertex (active), the external ear (reference), and the subcutaneous hind leg (ground), respectively. ABR click stimuli of different intensities (100 μsec duration) and tone pips (1 ms rise-fall time with 3 ms plateau) of 4, 8, 12, 16, 20, 24, 28, and 32 kHz frequencies were delivered using a Tucker Davis Technologies (TDT) workstation running SigGen32 software (Fetoni et al., 2018). Auditory function was tested by decreasing the sound intensities from 90 to 20 dB sound pressure level (SPL) in 5 dB SPL steps. The hearing threshold was determined by the lowest intensities at which reproducible electrical response waves could be recognized.

Distortion product otoacoustic emission response thresholds were tested as described previously (Men et al., 2015). The DPOAE responses at diction frequency 2f1-f2 were measured with two primary tone frequencies (f1 and f2 with f2/f1 = 1.2) to predict the auditory thresholds. DPOAE response thresholds were recorded at 8–32 kHz frequencies and intensities ranging from 90 to 20 dB SPL in 5 dB SPL decrements, the same as the ABR test protocol by the acoustic microphone probe and TDT system.

### Quantitative Real-Time Reverse Transcription PCR (qRT-PCR)

qRT-PCR was performed as previously described (Wang et al., 2017). Total RNA was extracted at 4°C using Trizol RNA isolating reagent (Thermo Fisher Scientific, Waltham, MA, United States) according to the established procedures. Total RNA (1000 ng) was reversely transcribed using PrimeScript^TM^ RT reagent kit with gDNA Eraser (perfect real time) kit (Takara, Japan) for the synthesis of a single-stranded cDNA library according to the manufacturer’s introduction. Gene-specific mRNA analyses were performed using the standard protocol of SYBR Premix Ex Taq^TM^ II (TliRnaseH plus) kit (Takara, Japan). Relative quantification was performed using the comparative threshold method (ΔΔCT) and *Gapdh* gene was used as a reference to normalize the specific gene mRNA expression. After amplification, each qPCR product was sequenced using electrophoresis to ensure the specificity. The primers used were listed in Table 1.

**TABLE 1:** List of qRT-PCR primer sequences.

Gene name	Primer sequence (5′-3′)	Length (bp)
*Fgf13*	F-CAGCCGACAAGGCTACCAC R-GTTCCGAGGTGTACAAGTATCC	184
*Fgf12*	F-GGAGAGCAAAGAACCCCAG R-CACCACACGCAGTCCTACAG	159
*Caspase 3*	F-GGAGCAGCTTTGTGTGTGTG R-CTTTCCAGTCAGACTCCGGC	131
*Caspase 9*	F-GGACCGTGACAAACTTGAGC R-TCTCCATCAAAGCCGTGACC	101
*Caspase 12*	F-CTGGCTCTCATCATCTGCAACAA R-CGGCCAGCAAACTGCATTAAC	173
*Cytochrome C*	F-GAGGCAAGCATAAGACTGGA R-TACTCCATCAGGGTATCCTC	133
*P53*	F-CCCGAGTATCTGGAAGACAG R-ATAGGTCGGCGGTTCAT	146
*Bcl-2*	F-TGACTTCTCTCGTCGCTACCG R-GTGAAGGGCGTCAGGTGCAG	69
*Bcl-xl*	F-GAGGCAGGCGATGAGTT R-ACGATGCGACCCCAGTTT	149
*Bak*	F-AAACCTCTCTCCCTACCCCA R-AGGATGGGGTTCAGTAGCAC	162
*Gapdh*	F-TGTCAGCAATGCATCCTGCA R-CCGTTCAGCTCTGGGATGAC	240

### Tissue Preparation

Mice were transcardially perfused with 4% paraformaldehyde (PFA) under terminal anesthesia (10% chloral hydrate, 0.04 mL/10 g body weight). Cochleae were quickly dissected, post-fixed at 4°C for 12 h, decalcified in 10% EDTA for 24–48 h and incubated in 10% and 30% sucrose solution, respectively, at 4°C for 24 h. The samples were then embedded in OCT, frozen, and cryosectioned in 10 μm-thick sections (von Bartheld et al., 2016). All middle sections of cochleae were used. For whole-mount immunostaining, cochleae were exposed to the sensory epithelium and dissected into basal, middle, and apical sections as previously described (Montgomery and Cox, 2016).

### Immunostaining Analysis and Confocal Imaging

After being washed by 10 mM PBS, specimens were permeabilized with 3% bovine serum albumin (BSA) and 0.3% Triton X-100 (Sigma, MO, United States) solution at 37°C for 1 h and blocked with 10% goat serum (Solarbio, China) at 37°C for 30 min and at RT for 30 min, respectively. For whole-mount staining, the cochleae were washed with 10 mM PBS and blocked (1% BSA, 1% Triton X-100 and 5% goat serum) at RT for 1 h. The sections or whole mounts were incubated with different primary antibodies: rabbit anti-FGF13 (1:200; Yenzym), mouse anti-tubulin βI- I- I (Tuj1; 1:200; GTX631836, GeneTex, United States), mouse anti-Myosin VIIa (1:200; sc-74516, Santa Cruz Biotechnology, United States), rabbit anti-cleaved caspase-3 (1:250; #9664, Cell Signaling, United States) and rabbit anti-cytochrome C (1:100; 10993-1-AP, Proteintech, United States) overnight at 4°C. The secondary antibodies were Fluorescein (FITC)-conjugated AffiniPure goat-anti-mouse IgG (1:300; 115-095-166, Jackson ImmunoResearch, United States) and cy^TM^ 3-conjugated AffiniPure goat-anti-rabbit IgG (1:200; 111-165-144, Jackson Immunoresearch, United States) diluted in the solution (1% BSA and 0.1% Triton X-100 for sections; 1% BSA, 0.1% Triton X-100 and 5% goat serum for whole mounts) at RT for 90 min. As controls for specificity, the primary antibody was co-incubated with the peptide used for immunization or samples were incubated with secondary antibody only. Nuclei were stained with 4, 6-diamidino-2-phenylindole (DAPI; 1:200; Southern Biotech, United States). The samples were observed under a laser scanning confocal microscope (Leica, Model: SP5, Wetzlar, Germany). For quantification of immunofluorescent staining, uniform microscope settings were kept during all image capture sections.

### Hematoxylin-Eosin Staining

The hematoxylin-eosin (H&E) staining was performed as described previously (Fetoni et al., 2018). Frozen-sections at a thickness of 10 μm were stained with hematoxylin and eosin for the histological assessment of cochlear morphological damage. A standard H&E protocol was followed with 3 min incubation in hematoxylin and 2.5 min staining in eosin, then mounted with neutral balsam (Solarbo, China). Only one middle section of a mouse cochlea was used to quantify the SGNs loss. SGNs were counted in the apical, middle, and basal regions of the cochlear sections using a × 20 objective as previously described (Someya et al., 2009). Type I and type II neurons were not differentiated, and viable neurons with a clear round nucleus and homogeneous cytoplasm were counted. The corresponding area of Rosenthal canal was measured on digital photomicrographs of each canal profile. The perimeter of the canal was traced with a cursor using ImageJ software (National Institutes of Health). The computer then calculated the area within the outline. The SGNs density was calculated as the number of SGNs per mm^2^.

### TUNEL Assay

TUNEL assay (in situ cell death detection kit, Roche) was used to examine DNA fragmentation in the nuclei of apoptotic cells in SGNs of the cochlea. The assay was performed on cochlear cryosections according to the manufacturer’s instructions. Specimens were permeabilized with 0.3% Triton X-100 and 3% BSA solution at 37°C for 1 h and incubated with freshly prepared working solution at 37°C for 2 h. After rinsing in 10 mM PBS, specimens were coverslipped. Nuclei of TUNEL-positive cells intensely labeled by lilac plus green were identified as apoptotic cells.

### Statistical Analysis

All data analyses were carried out with IBM SPSS 21 Statistics software and image processing with Origin 8, Adobe Illustrator 10 and GraphPad Prism 6.0. Data were presented as mean ± standard deviation ($\bar{x}$ ± SD). The comparisons between two groups were performed with the Student’s *t*-test and differences among groups were analyzed by One-Way ANOVA followed by Scheffe (C) and Bonferroni (B) analyses (SPSS). *P* < 0.05 was considered statistically significant.

## Results

### Localization of FGF13 in the Cochlea

**FIGURE 1:**
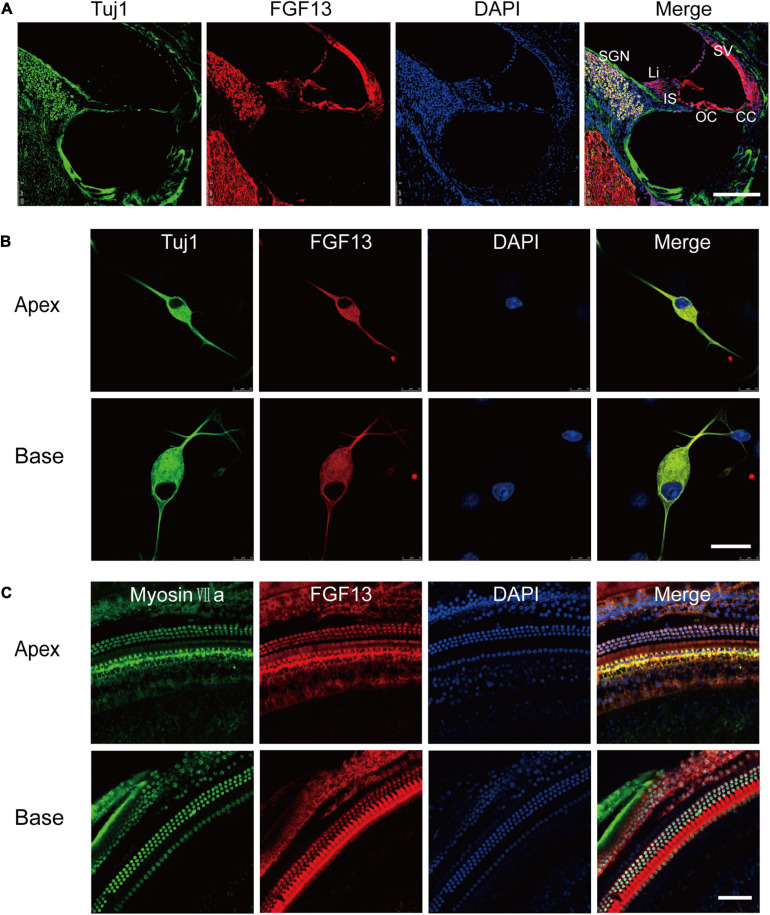
FGF13 is highly expressed in SGNs and HCs of the cochlea. **(A)** Confocal images of cryosections from the base of the cochlea show immunodetection of FGF13 (red), Tuj1 (green), and DAPI (blue). Tuj1 was used as a neuron marker. SV, stria vascularis; OC, organ of Corti; CC, Claudius’s cells; IS, inner sulcus; Li, spiral limbus; SGN, spiral ganglion neurons; Scale bar = 200 μm. **(B)** Immunofluorescence staining of cultured SGNs at a cellular level. Scale bar = 20 μm. **(C)** Confocal whole-mount images of FGF13 in hair cells. Myosin VIIa (green) served as a hair cell marker. Scale bar = 50 μm.

We first examined the localization of FGF13 in murine cochlear tissue using immunohistochemistry (IHC) (Figure 1). FGF13 was located primarily in the organ of Corti (OC), SGN, stria vascularis (SV), spiral limbus (Li), inner sulcus (IS), and the Claudius’ cells (CC) from the apical to basal regions of the cochlea tissue (Figure 1A). We further investigated the expression patterns of FGF13 in cultured SGNs and whole-mount HC staining. The results showed expression in the cytoplasm, membrane, and neurites of the SGN (Figure 1B). In HCs, staining was also observed in the cytoplasm and membrane, with more prominence in the inner rather than the outer regions (Figure 1C). We did not observe FGF13 expression in the nucleus of either the SGNs or HCs (Figures 1B,C). We did not find any significant difference between the post-natal expression levels in the mouse cochlea at P0, P7, P14, P30, and P60 days (Supplementary Figure 1).

### Generation of a Mouse Line With Conditional Knockout of *Fgf13* in the Inner Ear

**FIGURE 2:**
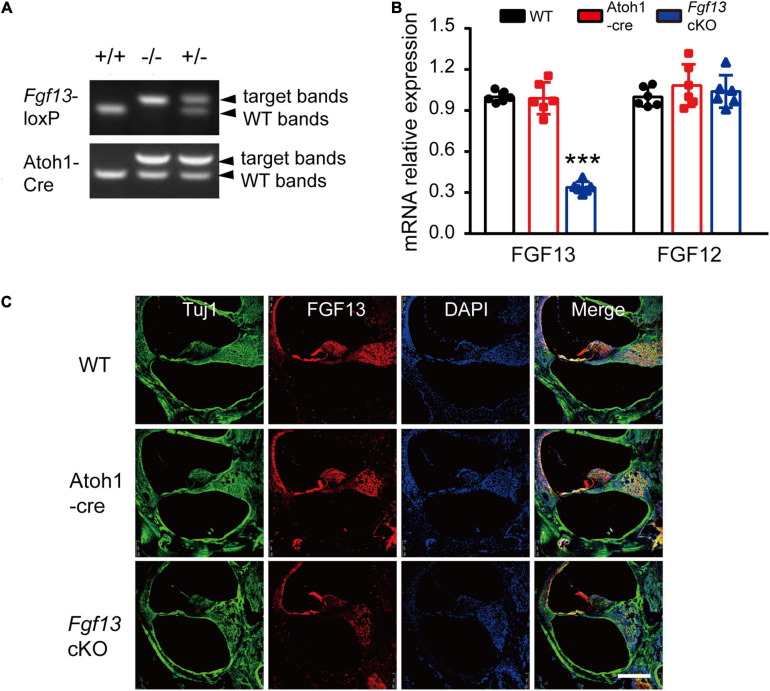
Generation of inner ear conditional knockout *Fgf13* mice using the *loxP-Cre* system. **(A)** PCR genotyping of wild-type (+/+), homozygote (–/Y or–/–), and hemizygous (+/–) *Fgf13* knockout mice using genomic DNA prepared from tail biopsies. **(B)** qRT-PCR of FGF13 and FGF12B. Expression levels were normalized to endogenous GAPDH. Each expression level was standardized to WT and given a value of 1. ***P < 0.001 compared to WT as determined by One-Way ANOVA. **(C)** Confocal images of FGF13 (red) and Tuj1 (green) in the base of the cochlea. Scale bar = 200 μm.

We generated a mouse line with a selectively deleted *Fgf13* in the inner ear using the *loxP/Cre* system, to investigate its role in auditory function. The gene is located on the X chromosome and comprises 72.3 kb of the genomic DNA in mice. Homozygous mice with floxed exon 3 of *Fgf13* (*Fgf13*^fl/Y^ or *Fgf13*^fl/fl^) were generated as described previously (Wang et al., 2017). To achieve inner ear-specific *Fgf13* deletion, *Fgf13*^fl/Y^ or *Fgf13*^fl/fl^ mice were crossed with Atoh1-cre mice (Matei et al., 2005). The latter consisted of a 1.5 kb *Atoh1* enhancer fragment that drove Cre expression. *Cre* mediated beta-galactosidase identified the *loxP* site, and was expressed in all HCs and SGNs, as well as some supporting cells of both the cochlea and the vestibule (Matei et al., 2005)^2^, resulting in the specific knockout of *Fgf13*. The pups were genotyped using PCR analysis (Figure 2A). To further confirm *Fgf13* specific deletion in the cochlea, quantitative reverse transcriptase PCR (qRT-PCR) was performed to determine the levels of cochlear mRNA in WT, Atoh1-cre, and *Fgf13* cKO mice. The gene transcript level was reduced to ∼33.8% in *Fgf13* cKO mice compared to levels in WT and Atoh1-cre control mice (Figure 2B). While FGF12 mRNA levels were unaltered in *Fgf13* cKO mice, confirming the specificity, efficacy as well as a lack of FGF12 compensation in the knockout (Figure 2B). Furthermore, IHC staining of the cochlea showed significantly decreased levels of FGF13 in *Fgf13* cKO mice (Figure 2C). Together, these data demonstrate the efficient deletion of *Fgf13* in the cochlea of the cKO mice.

### Elevated ABR Thresholds but Not DPOAE in *Fgf13* cKO Mice

**FIGURE 3:**
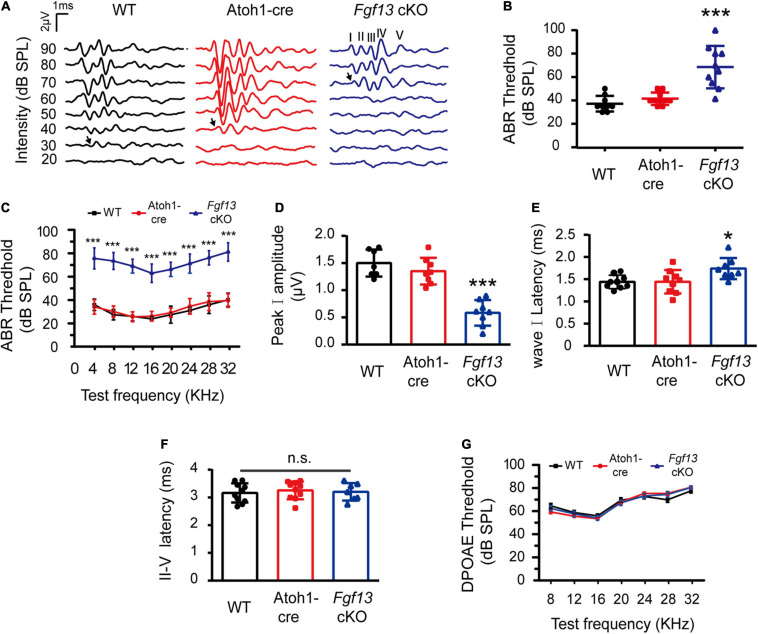
*Fgf13* cKO mice exhibit sensorineural hearing loss. **(A)** A representative click-evoked ABR waveform at decreasing intensities from 90 to 20 dB SPL in WT, Atoh1-cre and *Fgf13* cKO mice. Arrows indicate threshold levels. **(B,C)** ABR measurements for click **(B)** and tone burst responses **(C)**. *n* = 9/WT (5 M, 4 F); *n* = 10/Atoh1-cre (6 M, 4 F); *n* = 10/*Fgf13* cKO (7 M, 3 F). **(D–F)** The peak I amplitude **(D)**, latency of wave I **(E)**, and II-V **(F)** in ABR test were analyzed. The traces analyzed in **(D–F)** were from click test at SPL of 80 dB. *n* = 7/WT (5 M, 2 F); *n* = 8/Atoh1-cre and *Fgf13* cKO (5 M, 3 F) in peak I amplitude. *n* = 9/WT and Atoh1-cre (5 M, 4 F); *n* = 8/*Fgf13* cKO (5 M, 3 F) in wave I latency. *n* = 10/WT and Atoh1-cre (6 M, 4 F); *n* = 7/*Fgf13* cKO (5 F, 2 M) in II-V wave latency. *P < 0.05, ***P < 0.001 compared to controls as determined by One-Way ANOVA test. **(G)** DPOAE measurements in WT, Atoh1-cre and *Fgf13* cKO mice. *n* = 10/WT (6 F, 4 M); *n* = 9/Atoh1-cre (5 M, 4 F); *n* = 4/*Fgf13* cKO (3 M, 1 F). F, female; M, male; n.s., no significance. *P* > 0.05 as determined by One-Way ANOVA test.

We next sought to determine whether *Fgf13* cKO mice exhibited any hearing impairment-based phenotypes. The auditory brainstem response (ABR) test was performed to evaluate the functional integrity of the auditory system (Figure 3). The test consisted of a click stimulus in the 20–90 dB SPL range and frequency-specific stimuli for the tone test at 4–32 kHz. Consistent with our hypothesis, the *Fgf13* cKO mice displayed significantly higher ABR thresholds in response to the click stimulus and tone test across the entire auditory spectrum compared to WT and Atoh1-cre control mice, indicating the hearing impairment in cKO mice (Figures 3A–C). It is worth a mention that only the homozygous knockout mice (*Fgf13*^–/–^ or *Fgf13*^–/Y^) exhibited impaired auditory function, while the heterozygous mice (*Fgf13*^+/–^) showed a normal hearing threshold (Supplementary Figure 2). Further wave analysis in response to ABR click stimuli of 80 dB SPL revealed lower amplitude and increased latency of wave I (but not wave II-IV) in the *Fgf13* cKO mice compared to that of control groups (Figures 3D–F).

To test the function of the outer HCs, we measured the distortion product otoacoustic emission (DPOAE) test at frequencies of 8–32 kHz in the mice. Surprisingly, in comparison with WT and Atoh1-cre mice, no significant alteration of thresholds was found in *Fgf13* cKO mice at all tested frequencies, indicating the normal functioning of the outer HCs (Figure 3G).

### *Fgf13* Deficiency Reduced SGNs Density

**FIGURE 4:**
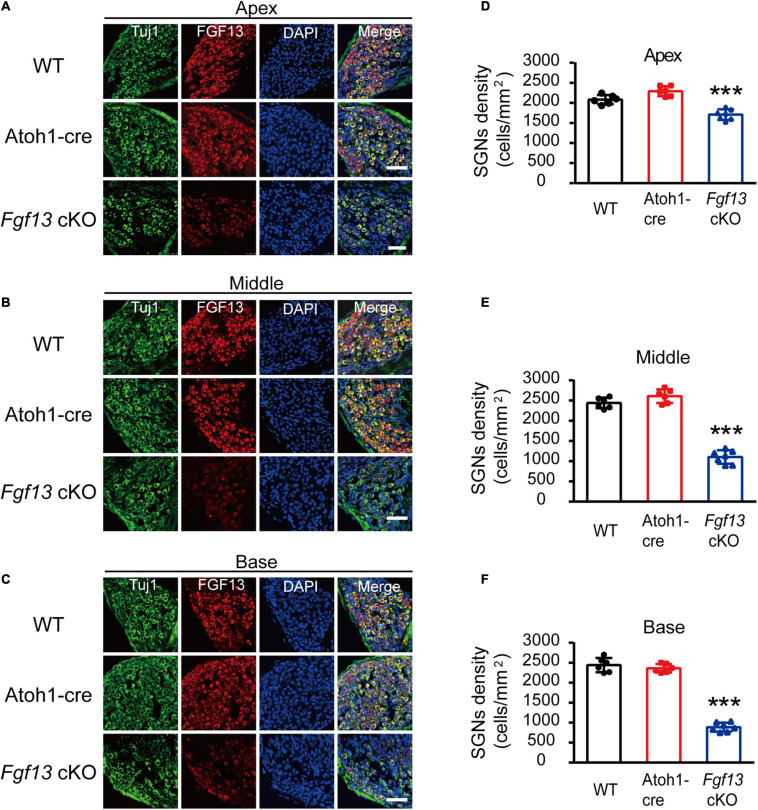
Reduced SGN density in *Fgf13* cKO mice. **(A–C)** Immunostaining of FGF13 in SGNs marked by Tuj1 from apex **(A)**, middle **(B)**, and base **(C)** in WT, Atoh1-cre and *Fgf13* cKO mice. Scale bar = 50 μm. **(D–F)** Quantitative assessment of SGNs densities from the apex to the base. *n* = 6/WT (5 M, 1 F); *n* = 6/Atoh1-cre (4 M, 2 F); *n* = 7/*Fgf13* cKO (5 M, 2 F). F, female; M, male. ***P < 0.001 compared to controls as determined by One-Way ANOVA.

The results for lower amplitude and increased latency of wave I in the ABR test suggested that *Fgf13* deficiency might cause damage to SGNs. Thus, we performed immunostaining on the SGNs to examine their morphology and any other changes in *Fgf13* cKO mice. Fluorescence intensity of FGF13 staining reduced significantly from the apex to the base in *Fgf13* cKO mice, confirming effective knockdown (Figures 4A–C). Importantly, when compared with controls, cell densities of type I SGNs (counts/mm^2^ marked by Tuj1, a neuronal marker) significantly decreased from the apex to the base (with normal morphology) in *Fgf13* cKO mice, with a more significant loss of SGNs in the base of the cochlea (Figures 4D–F).

**FIGURE 5:**
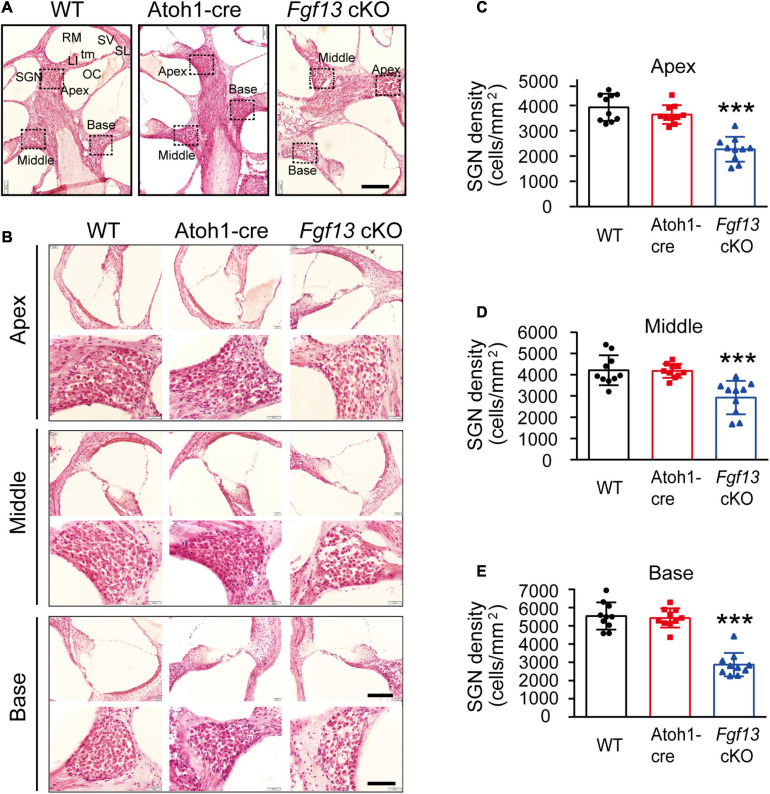
*Fgf13* knockout causes the loss of SGNs with normal morphological structure of the cochlea in the mice. **(A)** Modiolar cochlear sections depicting the apex to the base stained by hematoxylin-eosin. SGN, spiral ganglion neuron; LI, spiral limbus; OC, organ of Corti; tm, tectorial membrane; RM, Reissner’s membrane; SV, stria vascularis; SL, spiral ligament. **(B)** OC (top) and SGN (bottom) are shown at higher magnification. **(C–E)** Quantitative analysis of SGNs densities from the apex to the base. *n* = 10/WT (6 M, 4 F); *n* = 10/Atoh1-cre (7 M, 3 F), *n* = 10/*Fgf13* cKO (7 M, 3 F). Scale bar: G = 100 μm, Scale bar: H = 80 μm (top), and Scale bar: H = 40 μm (bottom). F, female; M, male. ***P < 0.001 compared to controls as determined by One-Way ANOVA.

**FIGURE 6:**
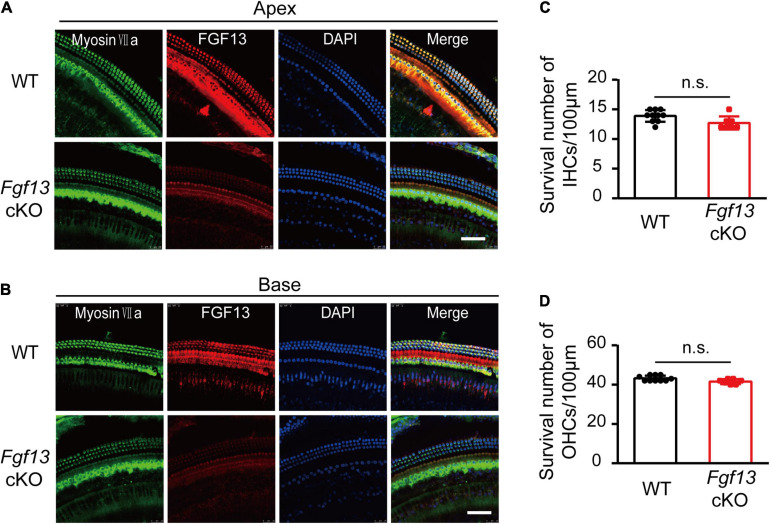
*Fgf13* cKO unalters the morphology and densities of HCs. **(A,B)** Confocal whole-mount images of cochlea HCs in WT and *Fgf13* cKO mice form the apex **(A)** to the base **(B)**. **(C,D)** Quantitative analysis of the survived inner HCs **(C)** and outer HCs **(D)**. There are no obvious abnormalities of morphology **(A,B)** and densities **(C,D)** for both inner HCs and outer HCs in *Fgf13* cKO mice. *n* = 10/WT (7 males, 3 females); *n* = 7/*Fgf13* cKO (4 males, 3 females). n.s., no significance. Scale bar = 50 μm; *P* > 0.05 as determined by One-Way ANOVA.

The gross histological features of the cochlear sections were also examined (Figure 5A). Hematoxylin-eosin staining data demonstrated that the cochlea of *Fgf13* cKO mice exhibited largely normal morphological structures of OC, SV, SL, and tectorial membrane with no obvious cell loss in either the inner or outer HCs (Figure 5B, upper panel). In contrast, loss of SGNs was observed in *Fgf13* cKO mice and was more significant in the basal region (Figure 5B lower panel, summary results in Figures 5C–E). Besides, whole-mount staining showed that the deletion of *Fgf13* did not affect the morphology and densities of both the inner and outer HCs from the apex to the base (Figure 6). These results were consistent with the normal functioning of the outer HCs as demonstrated by the DPOAE test (Figure 3G).

### Activated Mitochondrial Apoptosis Pathway in *Fgf13* cKO Mice

**FIGURE 7:**
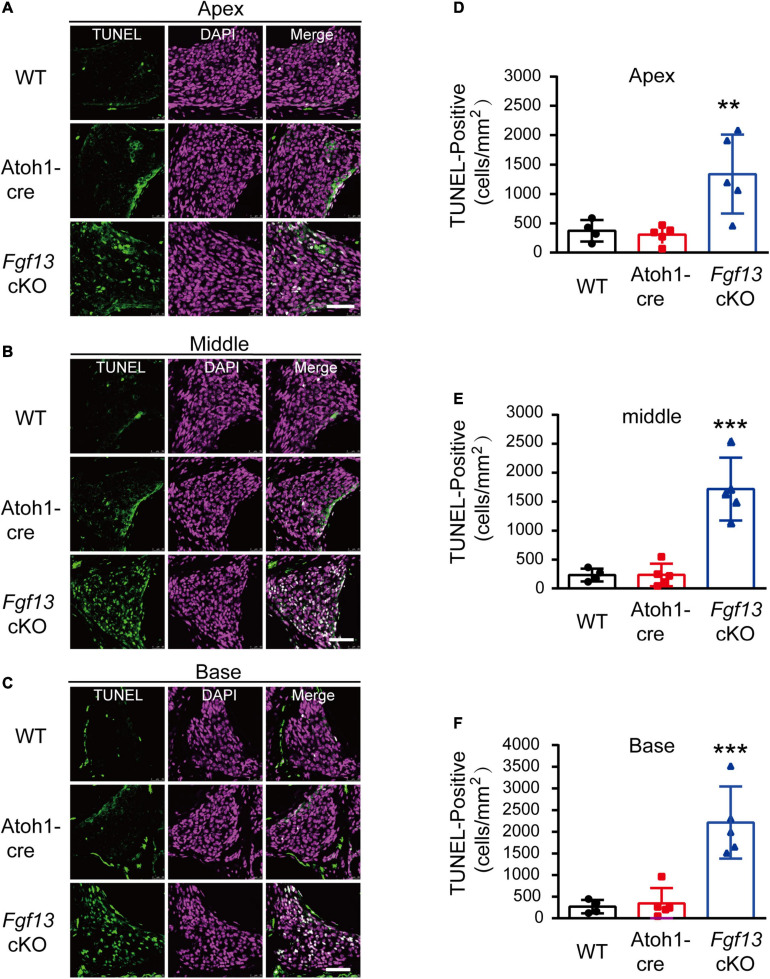
*Fgf13* cKO mice show accelerated SGNs apoptosis detected by TUNEL staining. **(A–C)** TUNEL staining of cochlea in WT, Atoh1-cre and *Fgf13* cKO mice. Scale bar = 50 μm. **(D–F)** Quantitative analysis of TUNEL-positive cells in apex **(D)**, middle **(E)** and base **(F)**. *n* = 4/WT (3 males, 1 females); *n* = 5/Atoh1-cre and *Fgf13* cKO (3 males, 2 females). **P < 0.01, ***P < 0.001 compared to controls as determined by One-Way ANOVA test.

**FIGURE 8:**
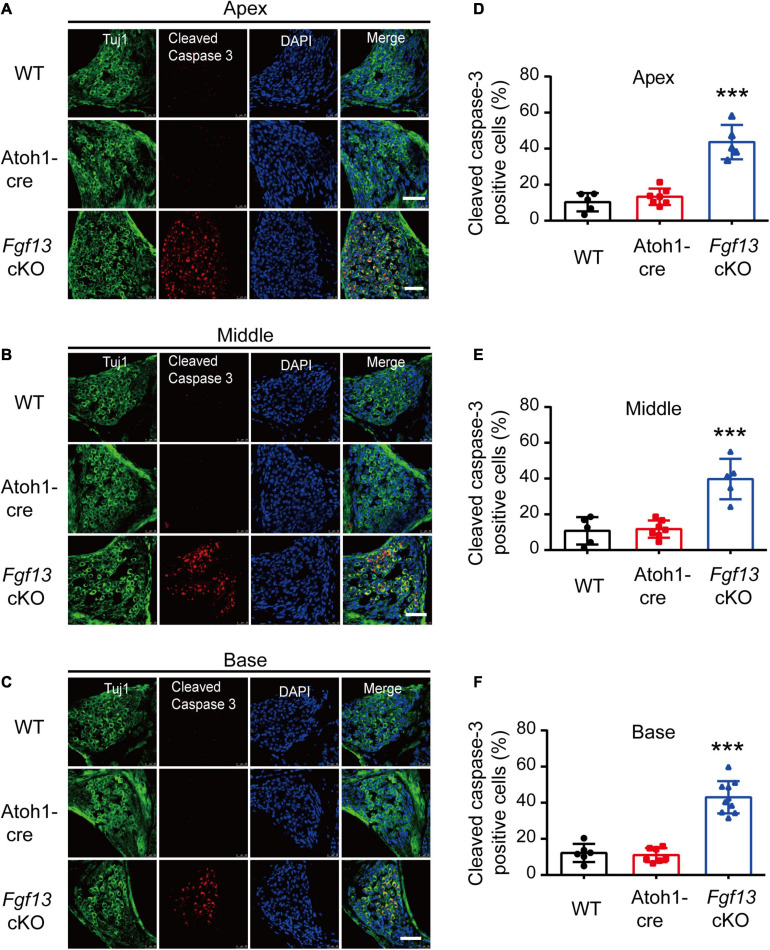
*Fgf13* cKO induces activation of cleaved-caspase-3 in SGNs. **(A–C)** The expression of cleaved-caspase-3 in cochlea SGNs from the apex to the base in WT, Atoh1-cre and *Fgf13* cKO mice. Scale bar = 50 μm. **(D–F)** Quantitative analysis of cleaved-caspase-3 positive cells in apex **(D)**, middle **(E)**, and base **(F)** for WT, Atoh1-cre and *Fgf13* cKO mice. Apex and Middle: *n* = 5/WT (4 M and 1 F); *n* = 7/Atoh1-cre (4 M, 3 F); *n* = 5/*Fgf13* cKO (3 M, 2 F). Base: *n* = 6 (5 M and 1 F) for WT, *n* = 7/Atoh1-cre (4 M, 3 F); *n* = 10/*Fgf13* cKO (7 M, 3 F). F, female, M, male. ***P < 0.001 compared with controls as detected by One-Way ANOVA test.

We further investigated whether the loss of SGNs in the cochlea was associated with apoptosis. We used the TUNEL assay to measure nuclear DNA fragmentation, a key feature of apoptosis (Martin et al., 2009; Someya et al., 2009). As shown in Figure 7, a significant number of TUNEL-positive SGNs were observed in *Fgf13* cKO mice from the apex to the base of the cochlea, with much fewer apoptotic cells detected in WT and Atoh1-cre mice. We further studied whether SGN apoptosis was caspase-dependent by examining caspase-3 activation through immunofluorescent staining with antibodies against cleaved-caspase-3 and Tuj1 (Figure 8). In agreement with the TUNEL results, the number of cleaved-caspase-3 positive cells was significantly higher in SGN of *Fgf13* cKO mice from the apex to the base compared to WT and Atoh1-cre controls (Figures 8A–C, summary results in Figures 8D–F), indicating the participation of caspases in the apoptotic pathway.

**FIGURE 9:**
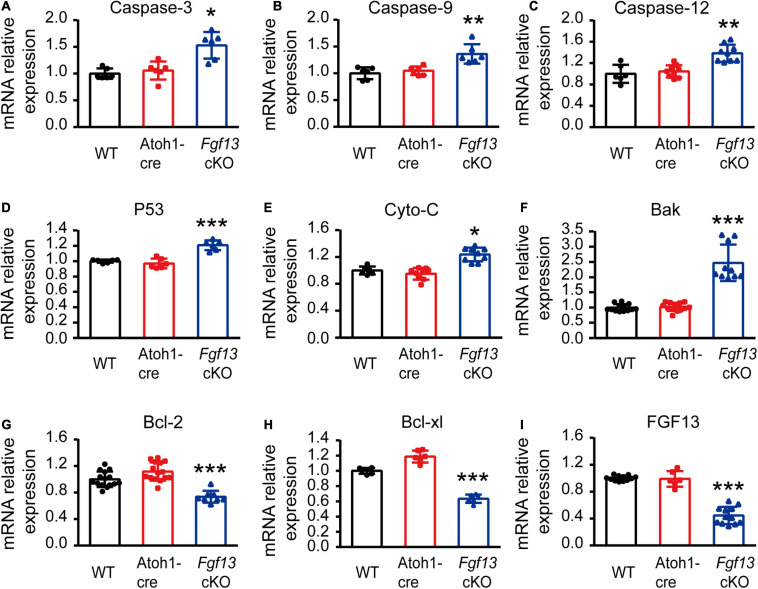
*Fgf13* deficiency changes apoptosis related gene expression through qRT-PCR. qRT-PCR data show significantly higher mRNA levels of caspase-3, caspase-9, caspase-12, P53, cytochrome C and Bak **(A–F),** and lower levels of Bcl-2 and Bcl-xl **(G,H)** accompanied by ∼53% reduction of FGF13 **(I)** in *Fgf13* cKO mice compared to the controls. Gene expression was calculated using the 2^–ΔΔCt^ method. Ct values were corrected for GAPDH and normalized to the WT group. *P < 0.05, **P < 0.01, and ***P < 0.001 compared with controls as detected by One-way ANOVA test.

**FIGURE 10:**
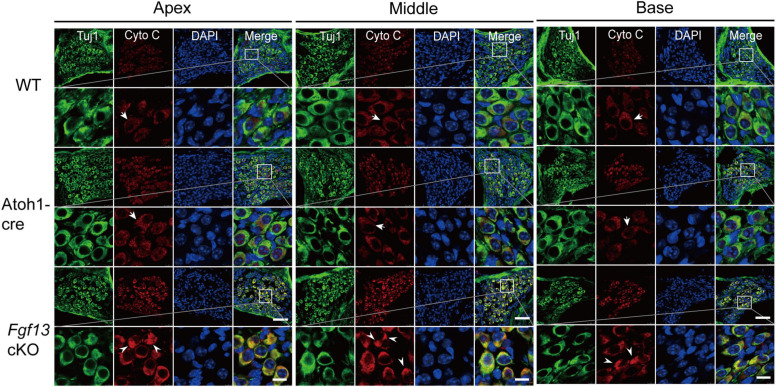
*Fgf13* cKO alters cytochrome C localization in SGNs. Immunofluorescence shows uniformly distributed cytochrome C (red) as puncta (arrows) from the apex to the base in SGNs of WT and Atoh1-cre mice. However, the distribution of cytochrome C was no longer uniform in the cytoplasm and are aggregated as a plaque (arrowhead) with increased expression in the cytoplasm of SGN in *Fgf13* cKO mice. Scale bar = 50 μm (top) and 10 μm (bottom).

To further investigate which pathways participated in SGN apoptosis, we examined the expression of several key factors in both extrinsic and intrinsic apoptotic pathways by qRT-PCR. *Fgf13* cKO mice exhibited significantly higher levels of pro-apoptotic genes, including *caspase-3*, *caspase-9*, *caspase-12*, *P53*, *cytochrome C*, and *Bak*, compared to that of WT and Atoh1-cre controls (Figures 9A–F). Consistently, the levels of anti-apoptotic factors *Bcl-2* and *Bcl-xl* were significantly decreased (Figures 9G,H) accompanied by a ∼53% FGF13 reduction in the cKO mice (Figure 9I). There were no significant alterations in *caspase-8*, *AIF*, *Bim*, and *Bax* among the three groups (Supplementary Figure 3), which indicates a potential activation of the mitochondrial apoptotic pathway (Wu and Bratton, 2013). Thus, we investigated the localization of cytochrome C, the release of which into the cytoplasm is a key feature for the activation of the mitochondrial apoptotic pathway. In both the WT and Atoh1-cre groups, cytochrome C was distributed uniformly as puncta in SGNs. In contrast, in *Fgf13* cKO mice, there was an uneven distribution as well as plaque aggregation accompanied by an increased expression in the cytoplasm of the neurons. These data indicate that knockout of *Fgf13* induced release of cytochrome C into the cytoplasm and hence, activated the mitochondrial apoptotic pathway in SGNs (Figure 10).

## Discussion

Multiple genes are involved in the development and functioning of the cochlea. They participate in various cellular functions and about 30% of congenital hereditary deafness has been reported to be SHL caused by genetic mutations (Ideura et al., 2019). For example, *Tbx1* gene mutations are associated with the human DiGeorge syndrome accompanied by a deficiency in hearing function (Chen et al., 2016). Moreover, functional null mutations of *Hars2* (encoding mitochondrial histidyl tRNA synthetase) cause Perrault syndrome, which is characterized by ovarian dysgenesis and sensorineural hearing loss (Pierce et al., 2011). Hearing loss is a common feature found in patients with BFLS, WS and CGH syndromes, all of which are characterized by genetic loss-of-function mutations in the *Fgf13* gene (Gecz et al., 1999; DeStefano et al., 2013; Abu-Amero et al., 2014). Whether the deafness in these patients is caused by the loss function of *Fgf13* and whether this gene plays any role in the auditory system is unknown. In this study, we found that selective deletion of *Fgf13* in the inner ear of mice caused sensorineural deafness. It also increased apoptotic cell loss of SGNs associated with the mitochondrial apoptotic pathway in the mouse cochlea. Our data revealed a novel role for *Fgf13* in the auditory system and suggest that the gene could be a potential candidate for understanding deafness.

In the current study, we investigated the role of *Fgf13* in hearing using the ABR and DPOAE tests. The ethological results showed that *Fgf13* cKO mice displayed impaired auditory function (Figures 3A–C) and gene deletion affected the wave I component but not the wave II-V in the click-evoked ABR test (Figures 3D–F). These results suggest that *Fgf13* deletion selectively causes damage to SGN but not to the central auditory pathway, since the amplitude and peak latency of wave I reflect the function of the peripheral SGNs, whereas waves II-V indicate a function of the ascending auditory pathway (Zuccotti et al., 2013; Eggermont, 2019). This observation was further supported by two results, abundant expression of FGF13 in SGNs of the inner ear (Figure 1) as well as that the cKO mice functioned normally in the DPOAE test (Figure 3G), indicating an intact function for outer HCs. Both SGNs and HCs play crucial roles in the production and transmission of sound. Although the investigation of the electrophysiological function of inner HCs and SGNs deserves further study, *Fgf13* cKO mice displayed normal morphology and densities of both inner and outer HCs, but showed significant cell loss in SGNs, indicating a contribution to hearing deficit in the knockout mice.

In previous studies, C57BL/6J mice developed a completely mature auditory pathway from P21 to P30 (Li et al., 2017), and showed a slow decline in auditory function, in a time-dependent manner, with age (Someya et al., 2009). Therefore, only mice aged 8–12 weeks were included in this study. In our study, 2-month-old *Fgf13* cKO mice showed sensorineural deafness and mitochondrial apoptosis associated with SGN loss, but the temporal aspects of the loss were unclear. RNA-seq results (Elkon et al., 2015) and online gEAR database showed a high expression level of FGF13 at E16, which reduced quickly until P0, and then remained stable at a low level at P7. The qRT-PCR results in our study also showed that the expression levels remained stable and no significant changes were observed between P0 and P60 in the mice (Supplementary Figure 1). This particular trend of FGF13 expression indicates a potentially important function in SGN development during the pre-natal stage. Atoh1-cre mediated beta-galactosidase caused *Fgf13* deletion in the otocyst as early as E10.5 (Matei et al., 2005). Moreover, *Fgf13* knockout only caused 30–47% SGN loss in 8–12-week-old mice, which might be increased gradually with age (Figures 4, 5). Due to this, we cannot exclude the possibility that *Fgf13* knockout could affect SGN development and display a congenital progressive hearing loss in mice, which would be an interesting topic for future studies (Ma et al., 2000; Pirvola et al., 2000).

SGN development is regulated by complex networks of transcription factors and signaling molecules, which have been proven in various transgenic mouse models (Kim et al., 2001; Puligilla et al., 2010; Yang et al., 2011; Dvorakova et al., 2016; Macova et al., 2019; Filova et al., 2020; Pavlinkova, 2020; Chizhikov et al., 2021). For example, *Neurod1* (the basic helix-loop-helix gene) is an essential gene for SGN development. *Neurod1* null mutants, aged 2–3 months, eventually lose most of their sensory neurons, cause projection defects of SGNs (Kim et al., 2001; Macova et al., 2019) and show the elevation at ∼35 dB of SPL at 4–38 kHz frequencies in the ABR test (Macova et al., 2019). In our study, *Fgf13* knockout mice aged 8–12 weeks showed 30–47% SGN loss and elevated ∼40 dB of SPL at 4–32 kHz frequencies in the ABR test, which might provide new perspectives on the molecular mechanisms underlying SGN loss and deafness. FGF13 is known to regulate neuronal polarization and migration by stabilizing microtubules. Loss of function results in an increase in the branching of axons and leading processes of cortical neurons (Wu et al., 2012). Therefore, we also cannot exclude the possibility that *Fgf13* knockout in the inner ear could affect the projection of SGNs, which may require future study to completely understand the role (Puligilla et al., 2007, 2010; Jahan et al., 2018).

Apoptosis is a major type of programmed cell death, where caspase activation plays a key role in the execution of the pathway. Caspases can be activated by two apoptotic pathways: the extrinsic (death receptor) pathway and the intrinsic (mitochondrial and ER stress) pathways (Li et al., 2017). We tested key signaling molecules associated with both pathways in *Fgf13* cKO mice and found higher levels of pro-apoptotic factors, which included caspase-9, cytochrome C, Bak, and lower levels of anti-apoptotic factors such as Bcl-2 and Bcl-xl (Figure 9). The uneven distribution of cytochrome C (Figure 10) and activated cleaved-caspase-3 (Figure 8) in *Fgf13* cKO mice indicated the involvement of the mitochondrial apoptotic pathway. The increased levels of caspase-12 suggested that ER stress may also be activated in the SGN of *Fgf13* cKO mice (Figure 9C). The detailed mechanisms underlying the activation of apoptosis require further study. A previous study showed that the downregulation of FGF13 elicited cell apoptosis directly through P53 interaction in cancer cells (Bublik et al., 2016). P53, a tumor suppressor, acts primarily as a transcription factor that regulates cell fate decisions, including cellular senescence, cell death, DNA repair, and metabolic homeostasis (Levine and Oren, 2009; Vousden and Prives, 2009). The activation of P53 can trigger apoptosis in a wide range of cell types, including neurons (Culmsee and Mattson, 2005). Expression of FGF13/miR-504 is repressed by P53, and depletion of FGF13 induces cell death by increasing P53 expression in cancer cells (Bublik et al., 2016). In our study, the higher expression of P53 mRNA in *Fgf13* cKO mice indicated the potential role of P53 in apoptotic SGN. It would be interesting to investigate whether knockout of *Fgf13* in SGN activates apoptosis through P53.

Bax and Bak belong to the multidomain Bcl-2 family of proteins, both of which are critically involved in the mitochondrial apoptotic pathway. In the present study, we observed higher levels of Bak mRNA with unaltered Bax mRNA levels in the cochlea of *Fgf13* cKO mice (Figure 8F and Supplementary Figure 3), indicating the participation of Bak in mitochondrial apoptosis but not Bax with suppression of *Fgf13*. These findings are also consistent with previous studies showing that Bak and Bax play different roles in mitochondrial apoptosis (Brooks et al., 2007; Fei et al., 2008; Someya et al., 2009). For example, Someya et al. (2009) showed that Bak-dependent (Bax-independent) mitochondrial apoptosis mediates age-related hearing loss in C57BL/6J mice. Thus, our data provides additional evidence for the specific function of *Bak* in mitochondrial apoptosis in the adult mouse cochlea.

## Conclusion

FGF13 is expressed predominantly in the OC, SGNs, SV, and supporting cells of the cochlear tissue. *Fgf13* conditional knockout in the inner ear induces sensorineural hearing loss, while also increasing the apoptotic cell loss of SGNs associated with the mitochondrial apoptotic pathway in the mice cochlea. Moreover, these findings reveal a novel role for *Fgf13* in hearing function and suggest that it could be a potentially novel candidate gene for understanding deafness. Thus, this study may provide new perspectives on the molecular mechanisms and novel therapeutic targets for deafness.

## Data Availability Statement

The original contributions presented in the study are included in the article/Supplementary Material, further inquiries can be directed to the corresponding authors.

## Ethics Statement

The animal study was reviewed and approved by Laboratory Animal Ethical and Welfare Committee of Hebei Medical University.

## Author Contributions

CW, YY, and JY designed the experiments. YY, JY, PL, RZ, QW, and ZD performed the research. WW, JT, GG, JS, and HZ contributed new reagents and analytic tools. YY, JY, and FL interpreted the data. YY, HZ, PL, and CW wrote and reviewed the manuscript. CW, HZ, and PL supervised the project. All authors contributed to the article and approved the submitted version.

## Conflict of Interest

The authors declare that the research was conducted in the absence of any commercial or financial relationships that could be construed as a potential conflict of interest.

## Footnotes

1 https://hereditaryhearingloss.org/

2 https://www.jax.org/strain/011104

## Abbreviations

ABR: auditory brainstem response
BFLS: BÖrjeson-Forssman-Lehmann syndrome
CC: Claudius’ cells
CGH: congenital generalized hypertrichosis
DPOAE: distortion product otoacoustic emission
FGF: fibroblast growth factor
FHF: FGF homologous factors
GEFS^+^: genetic epilepsy and febrile seizures plus
HCs: hair cells
IS: inner sulcus
Li: spiral limbus
NSHL: non-syndromic hearing loss
OC: organ of Corti
qRT-PCR: quantitative reverse transcriptase PCR
SGNs: spiral ganglion neurons
SHL: syndromic hearing loss
SPL: sound pressure level
SV: stria vascularis
WS: wildervanck syndrome
WT: wild-type.
