# Supplementary material for: Sensorineural Hearing Loss and Mitochondrial Apoptosis of Cochlear Spiral Ganglion Neurons in Fibroblast Growth Factor 13 Knockout Mice
Source: Front Cell Neurosci. 2021 Jun 16;15:658586. doi: 10.3389/fncel.2021.658586 (PMC8242186; doi:10.3389/fncel.2021.658586)
Supplement: Supplementary file 1 [file Data_Sheet_1.doc]

**Supplemental material**

**Figure S1 Stable mRNA levels of FGF13 in the mouse cochlea at P0-P60 days.**

The post-natal expression levels of FGF13 in the mouse cochlea at P0, P7 P14, P30, and P60 days examined by qRT-PCR. There were no significant differences among them. Each expression level was standardized to the WT and given a value of 1. *P* > 0.05 compared with controls as determined by One-Way ANOVA test.

**Figure S2 Heterozygous *Fgf13*^+/-^mice showed normal ABR threshold.**

ABR measurements for click **(A)** and tone burst responses **(B)** in WT, Atoh1-cre and *Fgf13^+/-^* mice aged 2 months. The number of mice used were n = 14 for WT, n = 15 for Atoh1-cre and n = 10 for *Fgf13^+/-^* mice. ABR thresholds of *Fgf13^+/-^* mice showed no significant difference compared to those of WT and Atoh1-cre controls in both click and tone test. *P* > 0.05 compared with controls as determined by One-Way ANOVA test. **Figure S3 Effect of *Fgf13* knockout in apoptosis related gene expression examined by qRT-PCR.**

qRT-PCR data showed that there were no significant differences in mRNA expressions of caspase-8, AIF, Bax and Bim **(A-D)** in *Fgf13* cKO mice compared to those of the control groups. The genes expression were calculated according to the 2^-ΔΔCt^ method. Ct values were corrected with GAPDH and normalized in the WT group. *P* > 0.05 compared to controls as detected by One-way ANOVA test.

Supplemental Table 1 Apoptosis related primers used in the experiments

| Gene name | Primer sequence (5’-3’) | Length (bp) |
| --- | --- | --- |
| *Caspase 8* | F-GCTGTATCCTATCCCACG  R-TCATCAGGCACTCCTTT | 181 |
| *Aif* | F-TGCTCTTGGCAGAAAGTCTC  R-TTGGGCATCACTTTCACTCC | 147 |
| *Bim* | F-CGACAGTCTCAGGAGGAACC  R-CCTTCTCCATACCAGACGGA | 195 |
| *Bax* | F-CGTGGTTGCCCTCTTCTACT  R-TTGGATCCAGACAAGCAGCC | 133 |
| *Gapdh* | F-TGTCAGCAATGCATCCTGCA  R-CCGTTCAGCTCTGGGATGAC | 240 |
