# Supplementary figures and images for: Sensorineural Hearing Loss and Mitochondrial Apoptosis of Cochlear Spiral Ganglion Neurons in Fibroblast Growth Factor 13 Knockout Mice
Source: Front Cell Neurosci. 2021 Jun 16;15:658586. doi: 10.3389/fncel.2021.658586 (PMC8242186; doi:10.3389/fncel.2021.658586)

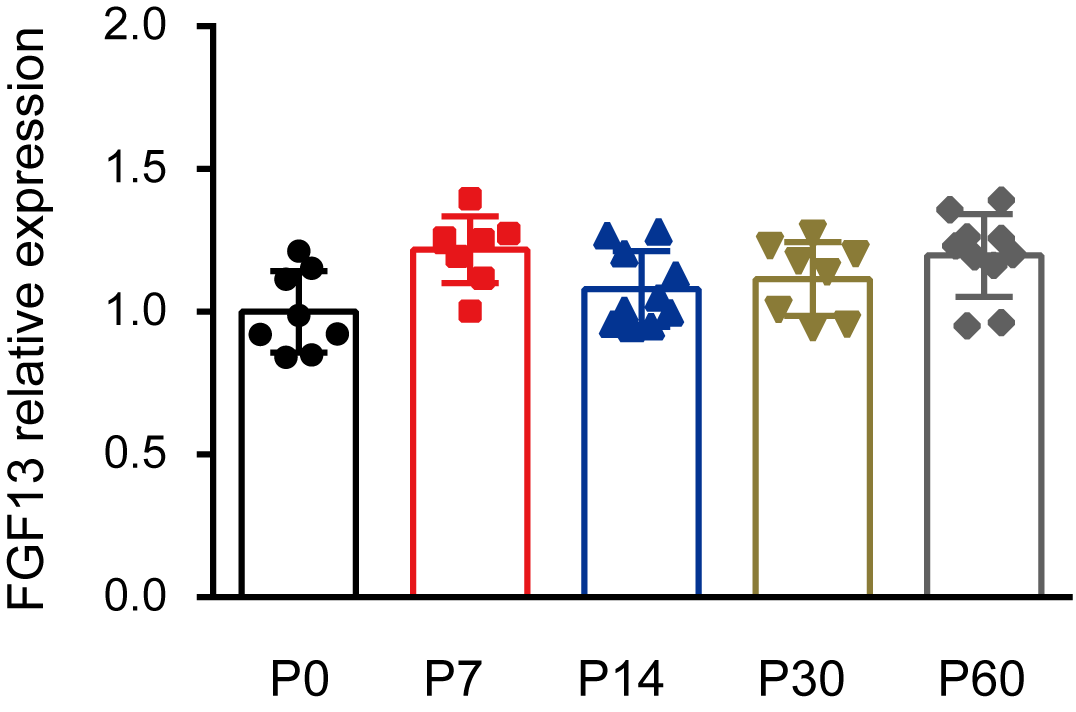

Supplement: Supplementary file 2 [file Image_1.TIF]

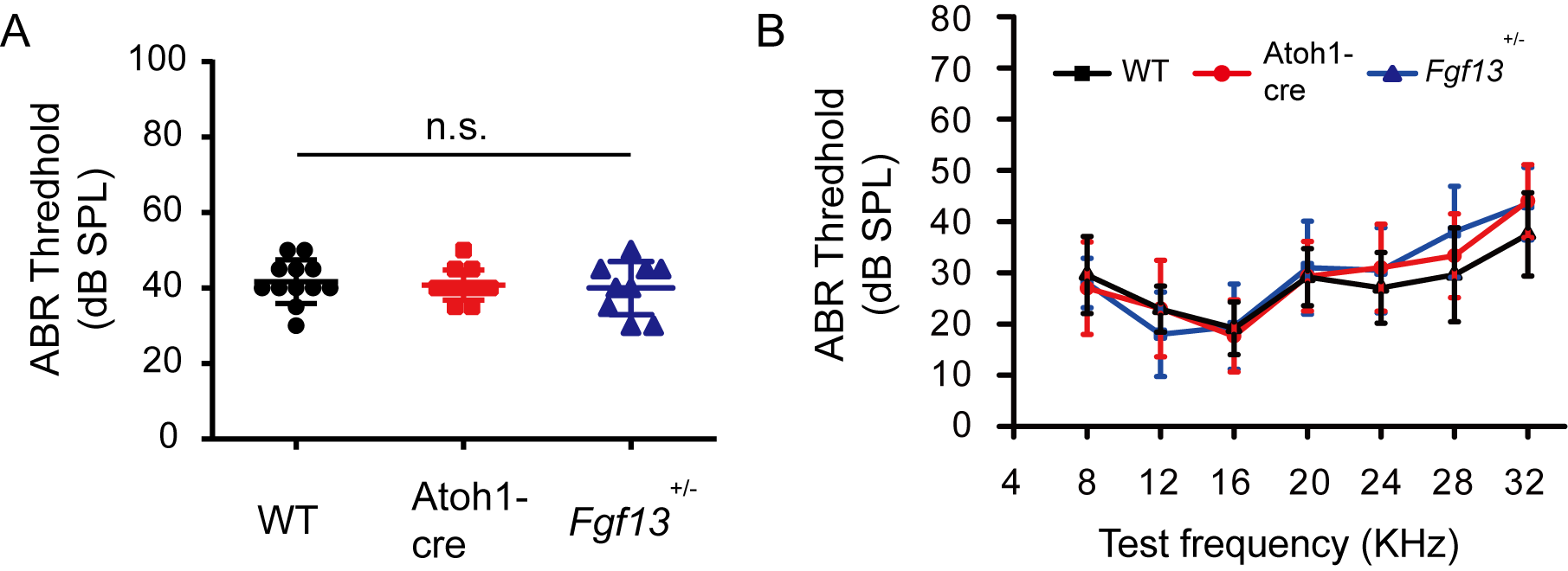

Supplement: Supplementary file 3 [file Image_2.TIF]

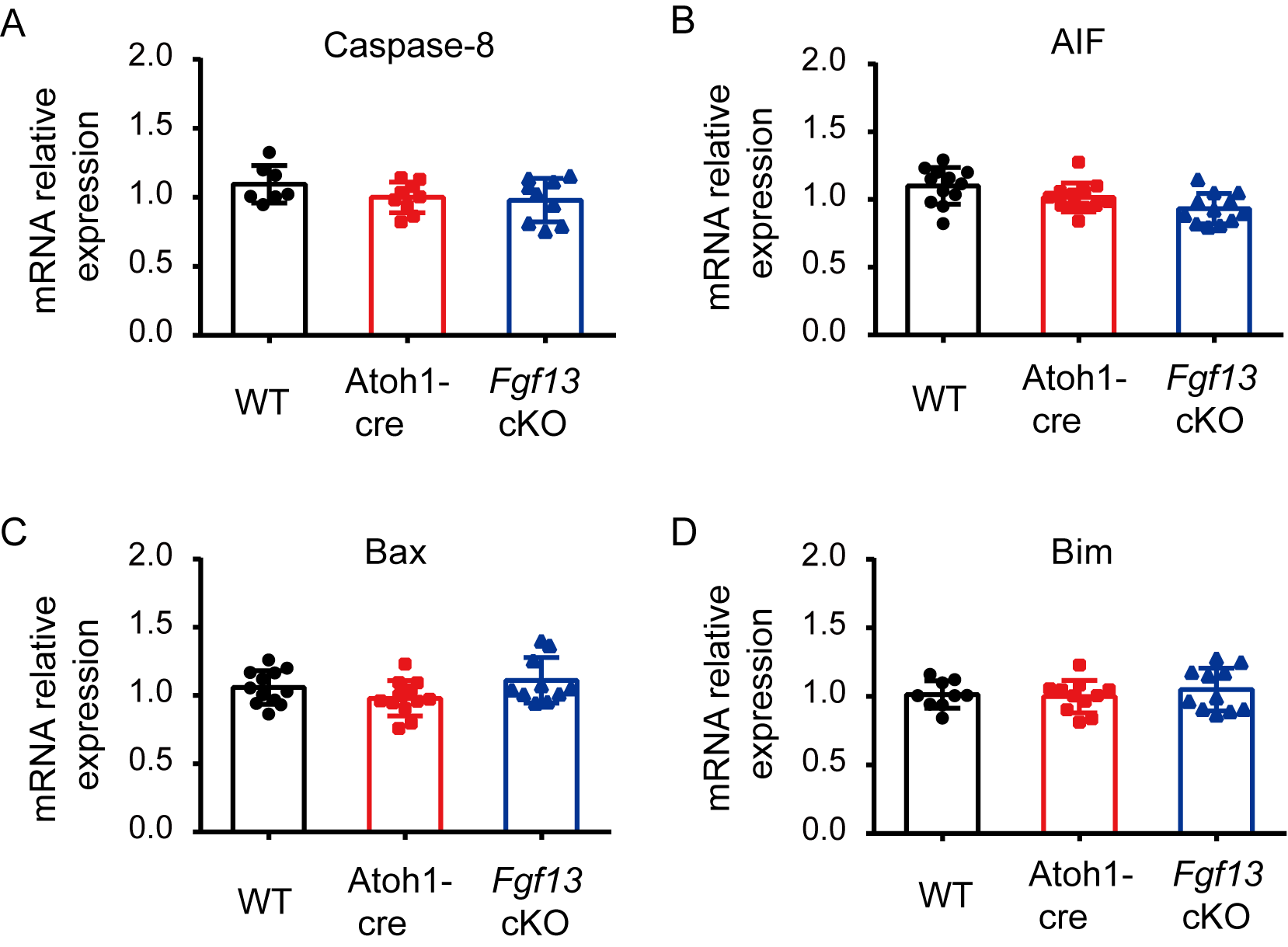

Supplement: Supplementary file 4 [file Image_3.TIF]
